# Spatial distribution of cerebral microbleeds reveals heterogeneous pathogenesis in CADASIL

**DOI:** 10.1007/s00330-021-08288-9

**Published:** 2021-10-26

**Authors:** Ya-Fang Chen, Chih-Hao Chen, Wen-Chau Wu, Bo-Ching Lee, Hsin-Hsi Tsai, Sung-Chun Tang

**Affiliations:** 1 Department of Medical Imaging, National Taiwan University Hospital, No. 7, Chung-Shan South Road, Taipei, 100 Taiwan; 2 Department of Neurology, National Taiwan University Hospital, No. 7, Chung-Shan South Road, Taipei, 100 Taiwan; 3 Institute of Medical Device and Imaging, National Taiwan University, No. 1, Sec. 1, Ren-Ai Road, Taipei, 100 Taiwan; 4 Graduate Institute of Clinical Medicine, National Taiwan University, No. 1, Sec. 1, Ren-Ai Road, Taipei, 100 Taiwan

**Keywords:** CADASIL, Magnetic resonance imaging, Microbleed, MRI, Small vessel disease

## Abstract

**Objectives:**

Radiological diagnosis of subtypes of cerebral small vessel diseases remains challenging. This study aimed to explore the spatial distribution of cerebral microbleeds (CMBs) in cerebral autosomal dominant arteriopathy with subcortical infarct and leukoencephalopathy (CADASIL) in contrast to cerebral amyloid angiopathy (CAA) in the lobar regions.

**Methods:**

Thirty-two patients with CADASIL and 33 patients with probable CAA were prospectively and consecutively included. On 3-Tesla susceptibility-weighted magnetic resonance images, CMBs were analyzed for incidence and volume within atlas-based regions of interest, followed by voxel-wise analysis using risk mapping. The distribution of CMBs was correlated with the status of hypertension. Correlation and group differences with a *p*-value less than 0.05 were considered to be significant.

**Results:**

As compared with the CAA group, the CADASIL group presents a larger CMB volume in hippocampus/amygdala and white matter (nonparametric analysis of covariance, *p* = 0.014 and 0.037, respectively), a smaller CMB volume in parietal lobe (*p* = 0.038), and a higher incidence in hippocampus/amygdala, white matter, and insula (logistic regression, *p* = 0.019, 0.024, and 0.30, respectively). As part of the exclusion criteria of probable CAA, thalamus, basal ganglia, and pons exhibit greater CMB volume/incidence in the CADASIL group. In CADASIL patients, hot spots of CMBs are identified in the putamen and posteromedial thalamus; hypertension is associated with larger CMB volumes in insula, basal ganglia, and pons.

**Conclusions:**

The spatial distribution of CMBs is differentiable between CADASIL and CAA in lobar regions. In CADASIL patients, hypertension has a region-dependent mediating effect on the CMB volume.

**Key Points:**

• *The topological distribution of lobar CMBs is differentiable between CADASIL and CAA.*

• *In CADASIL patients, hypertension mediates CMB volume and the mediation is region dependent.*

• *CMB risk mapping allows for voxel-wise exploration of CMB distribution and reveals hot spots in the putamen and posteromedial thalamus in CADASIL.*

## Introduction

Cerebral autosomal dominant arteriopathy with subcortical infarct and leukoencephalopathy (CADASIL) is a hereditary small-vessel disease (SVD) caused by mutations in the NOTCH3 gene that encodes a transmembrane receptor whose extracellular domain contains epidermal growth factor-like repeats [1]. In patients with CADASIL, the smooth muscle cells in the tunica media progressively degenerate, which leads to wall thickening, fibrosis, and lumen narrowing in the penetrating arterioles [2]. These changes have been associated with radiological manifestations such as white matter hyperintensity, lacunar infarct, and cerebral microbleed (CMB), although specific symptoms (e.g., migraine with aura, ischemic attack, and cognitive decline) and disease progression vary widely in patients [3].

CMBs are hemosiderin deposits in the brain that appear as punctate hypointense foci with a size of up to 10 mm on T_2_^*^-weighted or susceptibility-weighted magnetic resonance (MR) images [4]. In CADASIL, CMBs are found in 31–60% of the patients, more frequently in deep gray matter (e.g., thalamus) [5] but also in lobar regions [6]. On the other hand, lobar CMBs are a hallmark of cerebral amyloid angiopathy (CAA), another SVD characterized by β-amyloid deposits in the cortical and leptomeningeal arteries. However, radiological diagnosis of a specific type of SVDs may not be straightforward because aside from CMBs, SVDs can share imaging features such as lacune and white matter hyperintensity. Thus, before the result of molecular genetic testing and/or skin biopsy [7] is available and definitive, the presence of lobar CMBs can make differential diagnosis difficult. Understanding the topographic difference of CMBs between CADASIL and CAA may guide the clinicians to more precise patient workup and management, and may offer clues for investigation of the underlying pathophysiology. On the other hand, deep intracerebral hemorrhages (ICHs) have been associated with hypertension [8]. Whether the deep CMBs in CADASIL are mediated by hypertension remains to be elucidated.

In this study, we aimed to explore the spatial distribution of CMBs in CADASIL in contrast to CAA in the lobar regions. The distribution of CMBs was further correlated with the status of hypertension. We hypothesized that the spatial distribution of lobar CMBs in CADASIL is differentiable from that in CAA and mediated by hypertension.

## Materials and methods

### Subjects

Thirty-two patients with CADASIL (age = 61 ± 11 years; 11 women) and 33 patients with probable CAA (age = 77 ± 9 years; 21 women) were prospectively and consecutively included, as part of two ongoing CADASIL and ICH projects at National Taiwan University Hospital. The institutional review board approved this study. All subjects provided written informed consent before participation.

The diagnosis of CADASIL was based on genetically confirmed NOTCH3 cysteine-altering mutations. Patients were screened for NOTCH3 mutation when they had clinical and neuroimaging evidences suggestive of cerebral SVD. The process of genetic diagnosis included an initial p.R544C hot-spot mutation screening, followed by sequencing other most frequently reported NOTCH3 exons such as exon 3, 4, 5, 6, 11, or 18, if p.R544C mutation was not detected. The most common mutation point was p.R544C on exon 11 and was detected in 29 of the 32 patients. Probable CAA was diagnosed according to the modified Boston criteria [9] that require the subjects to be at least 55 years old and have hemorrhages restricted to lobar, cortical, or corticosubcortical regions. Patients with potential causes of hemorrhage including brain tumor, structural lesion, trauma, or medication were excluded. Table 1 summarizes the demographic and clinical characteristics of the patients.

**Table 1:** Demographic and clinical characteristics of the subjects. For age and symptom duration (both in units of years), group medians are reported with ranges shown in parentheses. *p*-values that remain significant after Benjamini–Hochberg correction with a false discovery rate of 0.05 are shown in boldface

	CADASIL	CAA	*p* value
Age (yrs)	60.5 (40–84)	78.0 (60–100)	** < 10**^**−5**^
Sex (female/male)	11/21	21/12	0.026
Diabetes	7 (22%)	5 (15%)	0.537
Hypertension	20 (63%)	22 (67%)	0.798
Hyperlipidemia	13 (41%)	7 (21%)	0.112
Smoking	10 (31%)	4 (12%)	0.076
Symptom duration (yrs)	3 (0.0–15.0)	0.7 (0.1–19.0)	**0.005**

### MR imaging

MR examinations were performed on two 3-Tesla whole-body clinical scanners (Tim Trio or Verio, Siemens) with the same imaging settings. The imaging protocol included T_1_-weighted three-dimensional magnetization-prepared rapid gradient-echo (voxel size = 1 × 1 × 1 mm^3^), fluid-attenuated inversion recovery, T_2_-weighted turbo spin echo, diffusion-weighted imaging (b-values = 0 and 1000 s/mm^2^), and susceptibility-weighted imaging (repetition time = 28 ms, echo time = 20 ms, voxel size = 0.8 × 0.8 × 1.6 mm^3^). The body coil was used to transmit radiofrequency pulses and a 12-channel phased-array head coil was used to receive signals.

### Data analysis

Image processing was performed using Statistical Parametric Mapping (www.fil.ion.ucl.ac.uk/spm) and customized programs in MATLAB (www.mathworks.com). Statistical analysis was performed using IBM SPSS Statistics for Windows, version 22 (IBM Corp.).

Two raters (Y.F.C., a licensed neuroradiologist with 20 years of experience, and C.H.C., a licensed neurologist with 5 years of experience) manually and separately defined CMBs on susceptibility-weighted images, and confirmed the results by consensus. Each subject’s susceptibility-weighted images were coregistered to her/his three-dimensional T_1_-weighted images. By using the DARTEL toolbox, the T_1_-weighted images of all the subjects included in this study were used to generate a study-specific template. The study-specific template was then normalized to the Montreal Neurological Institute template [10] (voxel size = 2 × 2 × 2 mm^3^), to which each subject’s CMBs were transferred by using the transformation matrices computed from the above steps.

In the Montreal Neurological Institute template’s coordinate system, regions of interest (ROIs) were created for 11 anatomical areas (as listed in Table 2) by using Automated Anatomical Labeling [11] and histogram-based segmentation. Two readers (Y.F.C. and W.C.W., a magnetic resonance physicist with 17 years of experience in medical image analysis) performed visual inspection on the overlay of CMBs and the anatomical ROIs to remove misassignment due to partial volume effect during spatial transformation. The volume and incidence of CMBs were calculated for each of the ROIs. Normalized CMB volume was also calculated by dividing the CMB volume within a given ROI by the corresponding ROI’s volume. Additionally, for each voxel the number of CMB-containing voxels within a ball of radius 5 mm was calculated. The number was used as an estimate of the risk of CMB at that location.

**Table 2:** CMB volume distribution in the Montreal Neurological Institute space. Group medians are reported in units of mm^3^ with ranges shown in parentheses. Group difference was examined by nonparametric analysis of covariance with symptom duration being the covariate. *p*-values that remain significant after Benjamini–Hochberg correction with a false discovery rate of 0.05 are shown in boldface. The italic rows are the regions included in the exclusion criteria of probable CAA

	CADASIL	CAA	*p* value
*Basal ganglia*	*112 (0–2160)*	*0*	** < *10***^***−3***^
*Thalamus*	*164 (0–3616)*	*0*	** < *10***^***−3***^
*Pons*	*32 (0–1240)*	*0*	** < *10***^***−3***^
Frontal lobe	12 (0–1648)	80 (0–16,248)	0.059
Parietal lobe	32 (0–2088)	112 (0–25,552)	0.038
Occipital lobe	24 (0–2088)	160 (0–12,616)	0.069
Temporal lobe	88 (0–2232)	208 (0–22,880)	0.058
Cingulum	0 (0–304)	0 (0–3128)	0.709
Insula	0 (0–272)	0 (0–2480)	0.075
Hippocampus/amygdala	20 (0–2456)	0 (0–2856)	**0.014**
White matter	172 (0–3136)	40 (0–5840)	0.037

Aside from CMBs, global white matter hyperintensity was assessed on fluid-attenuated inversion recovery images using the Fazekas scale (score = 0, 1, 2, 3, with 0 being absent and 3 the most severe) [12]. Mean apparent diffusion coefficient (ADC) was calculated in the aforementioned anatomical ROIs excluding the voxels that contained CMBs or ICHs.

The demographic and clinical characteristics were compared between groups (CADASIL vs. CAA) with Mann–Whitney *U* test or Fisher’s exact test when appropriate. A *p*-value less than 0.05 was considered to be significant. Multiple comparisons were accounted for using the Benjamini–Hochberg procedure with a false discovery rate of 0.05. The spatial distribution of CMBs was examined both on the ROI level and on the voxel level. On the ROI level, between-group difference was compared using non-parametric analysis of covariance for volumes and logistic regression for dichotomous dependent variables. On the voxel level, maps of CMB risk were calculated by averaging across subjects for each group, which was followed by permutation one-sample *t* test with a family-wise error rate of 0.05.

## Results

As shown in Table 1 and after correction for multiple comparisons, symptom duration (defined as the time between symptom onset and the MRI exam in this study) is longer in the CADASIL group (*p* = 0.005). The CADASIL group included younger patients (*p* < 10^−5^) as compared with the CAA group. After controlling for the effect of age, we were unable to find group difference in the spatial distribution of CMB measures. However, the age effect could predominantly come from the requirement for probable CAA patients to be 55 years or older in the Boston criteria. Including a covariate that is highly related to the grouping variable would remove considerable variance from the dependent variable and make the results meaningless [13]. In addition, we examined the correlation between age (the potential covariate) and the dependent variables (CMB measures in various anatomic regions) within each group, and no statistical correlation was found. Therefore, age was not considered in the following between-group analyses unless otherwise noted.

Tables 2 and 3 show the spatial distribution of CMB volume and incidence, respectively, controlling for symptom duration. As compared with the CAA group, the CADASIL group has a larger CMB volume in hippocampus/amygdala and white matter (*p* = 0.014 and 0.037, respectively), and a smaller CMB volume in parietal lobe (*p* = 0.038); the incidence is higher in hippocampus/amygdala, white matter, and insula (*p* = 0.019, 0.024, and 0.030, respectively). Among the above, CMB volume in hippocampus/amygdala remains significantly different between groups after correction for multiple comparisons. CMB volume and incidence are both highly different between groups in the thalamus, basal ganglia, and pons regardless of correction for multiple comparisons. This is because the presence of CMBs in these regions was part of the exclusion criteria of probable CAA.

**Table 3:** CMB incidence distribution. Group difference was examined by binomial logistic regression controlling for symptom duration. *p*-values that remain significant after Benjamini–Hochberg correction with a false discovery rate of 0.05 are shown in boldface. The italic rows are the regions included in the exclusion criteria of probable CAA

	CADASIL	CAA	*p* value
*Basal ganglia*	*24 (75%)*	*0 (0%)*	** < *10***^***−3***^
*Thalamus*	*20 (63%)*	*0 (0%)*	** < *10***^***−3***^
*Pons*	*18 (56%)*	*0 (0%)*	** < *10***^***−3***^
Frontal lobe	18 (56%)	22 (67%)	0.308
Parietal lobe	17 (53%)	25 (76%)	0.070
Occipital lobe	19 (59%)	27 (82%)	0.060
Temporal lobe	22 (69%)	27 (82%)	0.158
Cingulum	11 (34%)	11 (33%)	0.858
Insula	15 (47%)	7 (21%)	0.030
Hippocampus/amygdala	18 (56%)	9 (27%)	0.019
White matter	29 (91%)	21 (64%)	0.024

Hypertension was found to associate with larger CMB volumes in the insula, basal ganglia, and pons (*p* = 0.045, 0.023, and 0.015, respectively) in the CADASIL group, and in temporal lobe (*p* = 0.039) in the CAA group, according to nonparametric analysis of covariance with symptom duration as the covariate. Global Fazekas score was higher in the CADASIL group (*p* = 0.004) and moderately correlated with age in both groups (Spearman *ρ* = 0.412, *p* = 0.019 for the CADASIL group; *ρ* = 0.477, *p* = 0.005 for the CAA group). No group difference of ADC was found in any of the abovementioned ROIs.

**Fig. 1:**
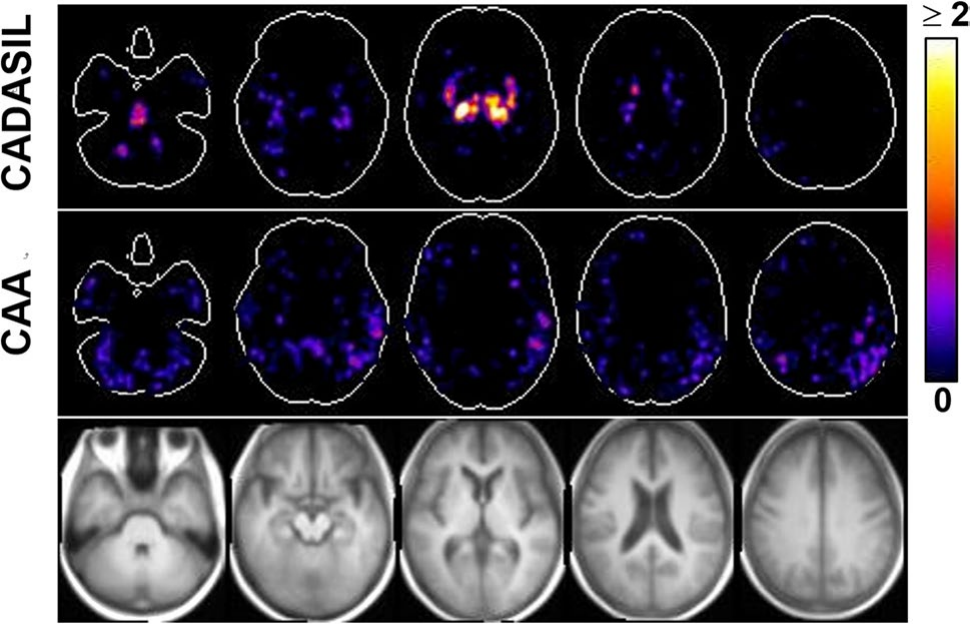
Maps of CMB risk. Five representative axial slices are shown. The color coding indicates the average count. Top row, CADASIL group. Middle row, CAA group. Bottom row, corresponding T_1_-weighted images

**Fig. 2:**
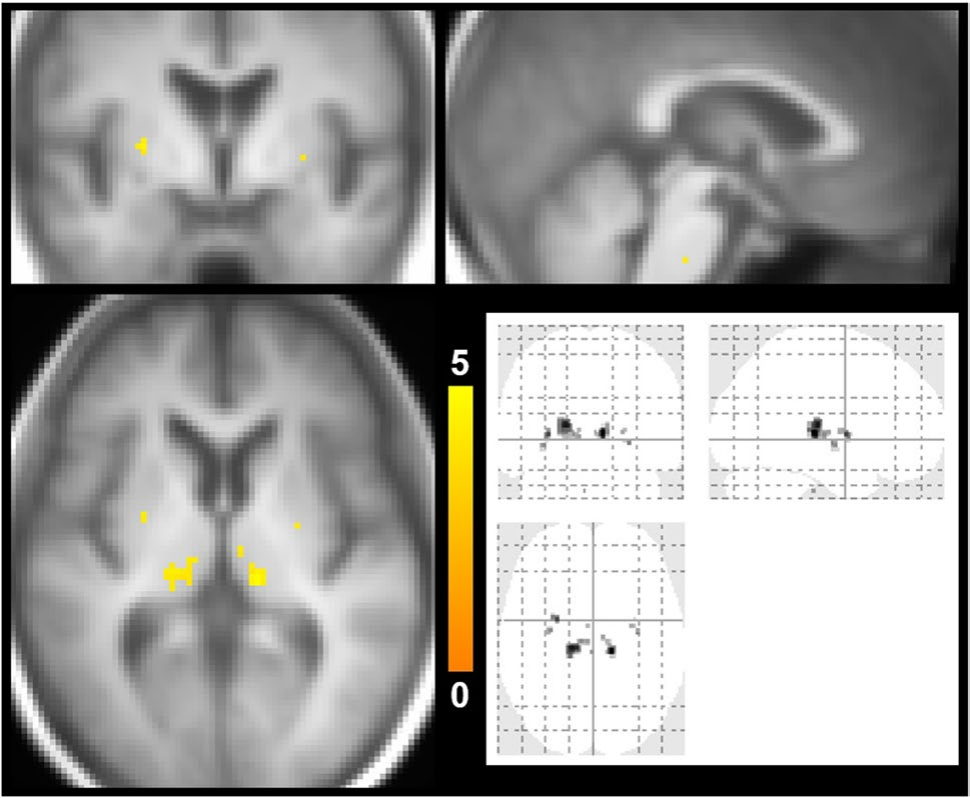
Statistical maps of CMB risk. In Fig. 1, the voxels that pass the one-sample Student *t* test at a family-wise error rate of 0.05 are highlighted (color coding indicates *t* value) and overlaid on T_1_-weighted anatomic images. Three representative orthogonal slices are shown. The bottom right panel displays the projection views

Figure 1 is the risk maps of CMBs. In the CADASIL group, hot spots are seen in the thalamus, basal ganglia, and pons. In the CAA group, counts are diffuse in parietal lobe and temporal lobe. One-sample Student *t* test at a family-wise error rate of 0.05 further revealed clusters in the thalamus, basal ganglia, and pons in the CADASIL group (Fig. 2), whereas no clusters were found in the CAA group.

**Fig. 3:**
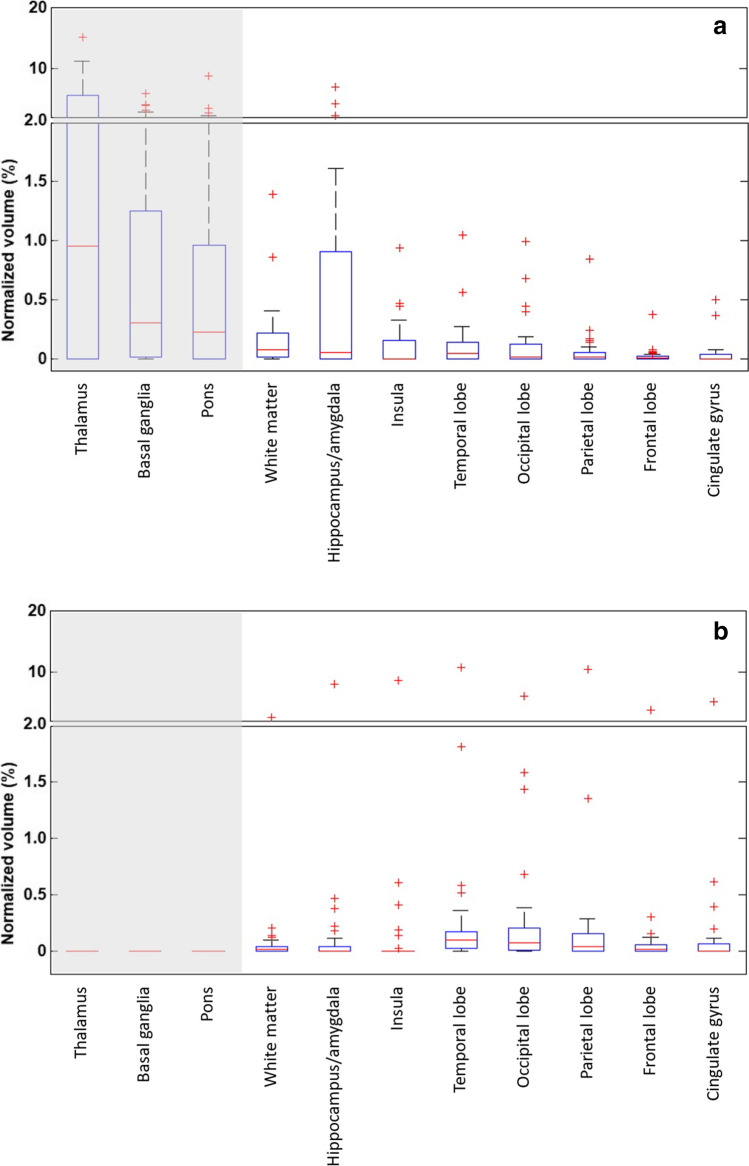
Box plots of CMB volume ratios. (**a**) CADASIL group. (**b**) CAA group. The lower and upper ends of the box indicate the first and third quartiles, respectively. The red line going through the box is the median. Maximum whisker = third quartile + 1.5 × interquartile range. Minimum whisker = first quartile − 1.5 × interquartile range. Shaded regions are the ones in the exclusion criteria for CAA

**Fig. 4:**
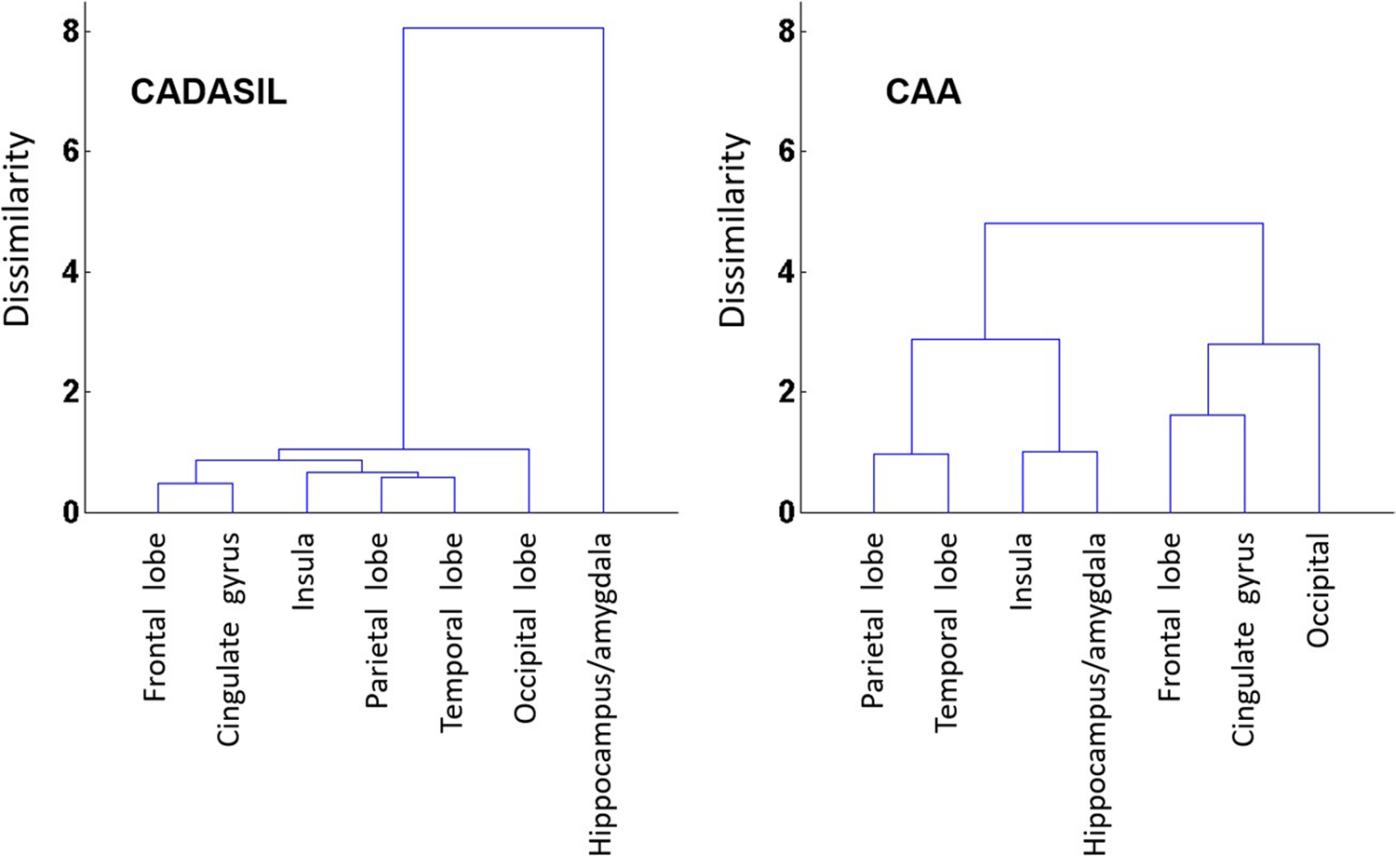
Hierarchical clustering of the lobar regions

Figure 3 shows the spatial distribution of normalized CMB volume (or volume ratio). No correlation was found between age or symptom duration and any of the volume ratios in either group, whereas in the CADASIL group, the volume ratio in insula was found to correlate with global Fazekas score (*ρ* = 0.490, *p* = 0.004). The lobar regions were then hierarchically clustered according to their CMB volume ratios. As shown in Fig. 4, the hippocampus/amygdala exhibits noticeable dissimilarity in the CADASIL group.

## Discussion

This study renders the following main results. First, CADASIL and CAA exhibited different lobar distribution of CMBs, in terms of volume, incidence, and inter-regional correlation. Second, hypertension was associated with the regions where CMBs occur in CADASIL. Third, hot spots of CMBs were identified in the putamen and posteromedial thalamus in CADASIL.

Although deep gray matter has been reported to be the preferential location of CMBs in CADASIL patients [5], as also observed in our data, lobar CMBs can occur and arouse concern about CAA. This particularly causes confusion when dealing with patients who present lobar hemorrhages along with lobar CMBs that are not abundant and superficial enough for an unequivocal impression of CAA. Further, some of these patients may have no deep CMBs or present prominent lobar CMBs along with few deep CMBs suggestive of comorbidity of CAA and hypertensive angiography. Indeed, more than 80% of the CADASIL patients in our study present lobar CMBs. Interestingly, these lobar CMBs exhibit different distribution patterns from those in CAA patients. In the parietal lobe, CMB volume is smaller in CADASIL patients (smaller in size and/or number) but the incidence is not statistically different between CADASIL and CAA. In the hippocampus/amygdala, both incidence and volume are larger in CADASIL patients. In the temporal/frontal/occipital lobes, the incidence and volume of CMBs in CADASIL patients are indistinguishable from those in CAA patients. These topographical patterns of lobar CMBs can help image-based differential diagnosis of CADASIL against CAA when CMBs are absent in thalamus/basal ganglia/pons, which accounted for approximately 12% of the CADASIL patients in our study.

The differential CMB distribution in hippocampus/amygdala and parietal lobe may account for the different manifestations of memory and cognition impairments in CADASL and CAA patients. Hippocampus plays a critical role in episodic memory and working memory [14]. Previous studies indicate that successful recollection of episodic information also requires activation of the parietal cortex [15] and that the frontal lobe and hippocampus both contribute to episodic memory performance [16]. Meanwhile, the parietal lobe has been found to associate with perceptual speed [17]. According to our data, CADASIL patients have larger CMB volume and incidence in the hippocampus, while CAA patients have larger CMB volume in the parietal lobe (the difference in the front lobe is just shy of significance). This could provide a plausible explanation for the deteriorated working memory but relatively preserved episodic memory in CADASIL patients [18]. On the other hand, declined perceptual speed and episodic memory are more commonly reported in CAA patients [19]. Nonetheless, the contribution of white matter cannot be ruled out. In our data, CADASIL patients tend to have more CMBs in white matter although our case number is not enough for further comparison among fiber tracts. It is also noteworthy that the existence of CMBs is not necessarily equivalent to cell damage. CMBs could have nonspecific influence as white matter hyperintensity.

In addition to the abovementioned group difference in CMB volume/incidence in corresponding regions, the inter-regional association also demonstrates differences between CADASIL and CAA groups (as shown in Fig. 4). Particularly in the CADASIL, the CMB volume ratio in the hippocampus/amygdala exhibits a noticeably dissimilar pattern with respect to other lobar regions. Taken together, the presence of CMB in the hippocampus/amygdala may distinguish CADASIL from CAA (i.e., between-group difference in CMB volume and incidence), and when associated with other lobar regions may suggest subtypes of CADASIL (i.e., its CMB volume ratio is notably large but cannot be inferred from other lobar regions).

Previous studies suggest different etiologies of lobar and deep ICHs, with the former contributed by amyloid toxicity and the latter being hypertensive. CMBs are different from ICHs in that they are local and smaller in size. In addition, CMBs are caused at least in part by dysfunction in blood–brain barrier (i.e., increased permeability leading to extravasation of red blood cells), whereas ICHs usually involve loss of vessel wall integrity. It is still unclear whether CMBs are precursors of primary ICHs or whether CMBs and ICHs have the same etiologies. Pathological examinations showed that CADASIL vessel damage is non-amyloid [1]. We showed that CADASIL CMBs also occur in lobar regions, with comparable incidence (53–59%) in frontal/parietal/occipital lobes. This suggests that lobar CMBs cannot be entirely explained by amyloid deposition and should have other causes. As for deep CMBs, our data showed high incidence in the thalamus (63%) and basal ganglia (75%), which reasonably agrees with previous studies [5, 20]. These regions appear to coincide with the venous tributary of the internal cerebral veins and basal veins which also drain the periventricular white matter, the main involvement area of arterial narrowing, white matter lesion, and lacunar infarct in CADASIL. Previous studies found collagenosis in the venules of CADASIL patients [21], which might be a reactive change to arteriopathy-associated microinfarct and/or inflammation. In addition, the medullary veins were reported to be fewer in number in CADASIL patients as compared with controls [22]. It is possible that deep cerebral venous dysfunction plays a role in the spatial distribution and/or pathogenesis of CMBs in CADASIL.

In this study, we introduced CMB risk mapping for voxel-wise exploration of CMB distribution. As compared with ROI-based analyses commonly used in previous studies, CMB risk mapping reveals hot spots of CMB in the putamen and posteromedial thalamus in CADASIL. In addition, we showed that hypertension increases CMB volume in the basal ganglia, insula, and pons. Although hypertension has been identified as a risk factor of CADASIL and other CVDs [23, 24], little is known about which anatomic regions are more susceptible to the deleterious factors (e.g., inflammation and oxidative stress) triggered by high blood pressure. Direct linkage between these regions and disease symptoms may not be straightforward however. Other contributing factors such as local vasculature (e.g., diameter and tortuosity) and cerebral blood flow [25] may need to be considered. Nonetheless, these findings may help further understanding of disease symptom and progression, and suggest heterogeneous pathogenesis in CADASIL.

This study has several limitations. First, the patient number is relatively small and dilated perivascular space (another radiological change recently found to correlate with cerebral SVDs [26]) is not assessed. Second, our data did not include complete battery of cognition tests. Whether alterations in cognition and memory in CADASIL can be related to region-specific CMB loads warrants further investigation. Third, aging-related brain atrophy could cause overestimation of CMB volume in the normalized space. Fourth, we did not include control subjects when examining the effect of hypertension. It would be informative to investigate whether the CMBs in healthy adults also occur in preferential regions. However, the subject recruitment would not be a straightforward task. The prevalence of CMBs in healthy adults was reported to be 6.4 [27] to 3.1% [28] according to population-based studies. Last but not least, the age effect was not controlled although to our knowledge, age dependence has not been reported regarding lobar regions of CMBs. Due to the inclusion criteria in the present study, the age difference between groups was so large that when controlling for it, no other group effects could be found. However, this could be due to the small patient number as well as the narrow age range in each group.

In conclusion, this study has shown differential spatial distribution of CMBs between CADASIL and CAA in lobar regions. In CADASIL patients, hypertension was found to mediate CMB volume and the mediation was region dependent.

## Abbreviations

CAA: Cerebral amyloid angiopathy
CADASIL: Cerebral autosomal dominant arteriopathy with subcortical infarct and leukoencephalopathy
CMB: Cerebral microbleed
SVD: Small vessel disease

## Declarations

### Guarantor

The scientific guarantor of this publication is Wen-Chau Wu.

### Statistics and biometry

No complex statistical methods were necessary for this paper.

### Methodology

• prospective

• cross sectional study

• performed at one institution
